# Tertiary students maintaining control over depression, anxiety, and stress during the pandemic—An emerging market perspective

**DOI:** 10.3389/fpsyg.2022.990192

**Published:** 2022-09-29

**Authors:** Larisa Ivascu, Benedict Valentine Arulanandam, Alin Artene, Prema Selvarajah, Lim Fung Ching, Chitra Devi Ragunathan

**Affiliations:** ^1^Faculty of Management in Production and Transportation, Politehnica University of Timişoara, Timişoara, Romania; ^2^Sunway College, Kuala Lumpur, Malaysia

**Keywords:** higher education, depression, anxiety, stress, coping strategies, self-efficacy, strategy, student

## Abstract

The higher education sector was affected by this pandemic, managing enduring challenges since early 2020. Institutions of higher learning (IHL) are prepared to address unsurmountable challenges to ensure that students are not deceived and are being given the proper nurture, coupled with adherence to syllabuses. Simultaneously, the COVID-19 pandemic has caused unscrupulous pressure on students of these institutions. The psychological waves are creating mammoth consequences, affecting the beneficiaries of the higher education system and their families. In recent years, with limited studies on psychological impact among tertiary students on a cross-country basis, general self-efficacy, and the degree of coping strategies, we were motivated to investigate the degree of depression, anxiety, and stress (DAS), among this cohort of students encompassing the pre-university/Diploma, 1st–4th-year undergraduate, and postgraduate students from private universities in Malaysia, Indonesia, India, Southern Africa, and China, representing the emerging economies. A cross-sectional survey was conducted, followed by quantitative analysis. The objective of this study was to recognize whether there is a relationship between the psychological impact of DAS and the coping strategies adopted by the undergraduate students responding during the lockdown. The findings of this study revealed that with a sample size of 397, DAS lacked any severe impact on students across gender, country, household income, and level of education. DAS was established to be well managed with a coping strategy and self-efficacy established. This study resulted in a deeper understanding of DAS among undergraduates in emerging economies and their degree of coping behavior, providing a glimpse of the approach of millennials to handle DAS during the pandemic.

## Introduction

The COVID-19 pandemic introduced a significant and profound impact on global society and affected the very fabric of society, leaving irreconcilable scars. The incidence of increased mortality and the mental stress condition of societies cannot be ignored. Population general health is something worth making progress toward. At different stages, the health propensities that are best for individuals will change when the life of these individual changes. Wellbeing must be drawn nearer in a comprehensive manner that considers both physical and mental. According to the World Health Organization [WHO] (2020), health is defined as “a state of complete physical, mental, and social wellbeing and not merely the absence of disease or infirmity.” Thus, mental health is crucial as it affects emotions and leads to how we feel, think, and act. According to WHO (2021), depression is a common mental disorder, and it is estimated that 5% of adults suffer from depression. University students are a special group of people who are at the peak of the transition period from initial adulthood to adulthood which can be one of the most challenging periods in one’s life. New campus environment, fresh cohort of students, tight assignment deadlines, mastering new skills, away from family, and uncertain job opportunities cause anxiety for students which contributes to stress and depression. Holmes et al. (2020) posit that this pandemic has brought about considerable mental health issues. Prior to this study, there were numerous studies highlighting the issue of a high surge in mental health (Ibrahim et al., 2013; Rotenstein et al., 2016).

Studies on depression, anxiety, and stress (DAS) have had mixed results. Studies by Thombs et al. (2020) indicated that in a longitudinal study, it was found that there was a small to negligible increase in mental health issues. Simultaneously, studies by Fancourt et al. (2021) highlighted that there were decreasing levels of anxiety and depression during the beginning period of the lockdowns in the United Kingdom. A similar surprise was found in a study by Fried et al. (2020), in which there were decreasing levels of depression, anxiety during the COVID-19 pandemic, contrary to the studies by Elmer et al. (2020) and Huckins et al. (2020) which reported that such levels were increasing during the pandemic. Similarly, a study by Fawaz and Samaha (2021), using quantitative methodology, involving 520 Lebanese undergraduate university students found that in an E Learning environment, there was an increase in DAS. Further studies by Baloran (2020), Capone et al. (2020), Lai et al. (2020), Nurunnabi et al. (2020), Wathelet et al. (2020), Almomani et al. (2021), Jiang et al. (2021), Mekonen et al. (2021), Patias et al. (2021), Sood and Sharma (2021), and Villani et al. (2021) postulate that the COVID-19 pandemic has resulted in mental and emotional disorders among tertiary students, leading to behavior abnormalities and health challenges. An additional study by AlAteeq et al. (2020) reported females and university students showed significant association with stress level. Luke et al. (2021) conducted a similar survey and proposed that depression, stress, and anxiety are extremely prevalent among university students even after the lockdown was lifted. However, in another student involving 1,836 Swedish university students, concluded that the level of DAS indication was steady during the first 3 months of the pandemic but declined during the summer months. In their studies (Cioca et al., 2019; Mohsin et al., 2022), they show the importance of investigating personal factors on student development. Furthermore, our study employs the general self-efficacy scale (Schwarzer and Jerusalem, 1995), as a moderator to identify the strategies that exist that are intended to mitigate the level of DAS. These influences are felt in the academic environment and in the business environment.

This research topic is limited in Malaysia and lacks information regarding the involvement of university students on a cross border sample size, and using general self-efficacy scale with coping strategies, the authors of this study found that it necessitates to discover the level of DAS among university students in Malaysia and emerging markets during this pandemic. The aim of this study is to recognize whether there is a relationship between the psychological impact of DAS and the coping strategies adopted by the university students during the lockdown. Aside from this, our study employs the general self-efficacy scale (Schwarzer and Jerusalem, 1995), as a moderator to identify the coping strategies which are intended to mitigate the level of DAS. In addition, this study engages the social cognitive theory (Bandura, 1986, 2008), as its theoretical stance, which holds that environment, cognition, and behavior are key influencers on the belief system of an individual. In social cognitive theory, self-efficacy is seen as a source with respect to stress vulnerability. Therefore, an event can be viewed as negative only after a negative cognitive assessment.

The pandemic period requires conducting studies on the impact of DAS factors on student development. This research investigates the relationship between the psychological impact of DAS and the coping strategies adopted by the research respondents during the lockdown period. This period is essential for students and develops traces on their professional development. This study would result in a deeper understanding of DAS among tertiary students in emerging economies and their degree of coping behavior, which will allow regulators and institutions to better address DAS in institutions of higher learning (IHL), coupled with prudent mitigation actions. This work contributes to good decisions in higher education and to the expansion of research carried out during the pandemic by presenting the results obtained.

## A global overview of the COVID-19 pandemic

The World Health Organization (WHO) notified on 27 March 2020, at the start of the COVID-19 pandemic, that stress, anxiety, and fear would increase the result of this negative circumstance, which would cause uncertainty and concern among people of all ages (World Health Organization [WHO], 2020). To fight the onslaught of this deadly virus, the lockdown that followed the unexpected outbreak had turned people from social “creatures” to isolated humans. Research on the COVID-19 pandemic by Sifat (2020) revealed that suicide, domestic violence, mental disorders, anxiety, and depressive disorders were increasing in most countries. The pandemic continued to report new cases of infection as well as a concerning increase in the number of fatalities worldwide (Taucean et al., 2019; Sundarasen K. et al., 2020).

The higher education system has been affected by the COVID-19 pandemic. Higher education institutions, colleges, schools, and pre-schools in most affected countries resorted to online learning, which is a new learning experience for many students of varying ages (Sifat, 2020). Some psychological problems were subsequently reported, such as anxiety, depression, frustration, and trauma affecting students due to strict physical and social distancing in most countries and in more severe instances, total isolation. The adverse impact on students had impacted the entire teaching-learning environment, as evidenced in the literature in many countries and regions (UNESCO, 2021).

In the United States (US), Browning et al. (2021) in their recent research on psychological impacts among university students across seven states in the US reveal that university students are increasingly being recognized as a vulnerable population who suffer from higher levels of anxiety, depression, and even substance abuse and eating disorders as compared to the general population.

In contrast to similar findings across many countries as discussed above, Johansson et al.’s (2021) research across six universities in Sweden revealed a different set of findings. Using a DASS-21 data collection tool, which is a reputed measurement tool for all psychometric variables, thus limiting the risk of misclassification, the scholars conducted a prospective sample involving 1,836 full-time final-year undergraduates to conduct a cohort study on DAS before and during 6 months of the COVID-19 pandemic.

Interestingly, The Lancet (2020) and Fancourt et al. (2021), using a GAD tool with a large analytical sample of 36,250 undergraduates in UK universities in various stages of studies, revealed a different set of findings. The data suggest that high levels of depression and anxiety occurred in the early stages of lockdown.

## The psychological impact of COVID-19 in a Malaysian context

Malaysia has been dealing with the threat of COVID-19 since the appearance of the first cluster, which was discovered on 24 January 2020, results that have been published internationally (Hamid et al., 2021; Ramli and Jamri, 2021). Despite numerous lockdown measures used by the government to control the numbers, the number of COVID-19-positive patients has since continued to increase at an astounding rate. Since mid-2020, the majority of IHL campuses in Malaysia have prohibited face-to-face instruction to slow the pandemic’s spread. Instead, instruction is now largely delivered online. Although methods including lockdown, tight isolation, social separation, and remote emergency teachings have primarily stopped the development of COVID-19 in Malaysia, Sundaresan V. et al. (2020) claim that there is still room for improvement.

Basic demographic information such as gender, age, name of the institution, field of study, level of study, year of study, nationality, ethnicity, current mode of education (virtual or online), and Students’ living circumstances was among the research instruments employed in this study. Zung’s self-rated anxiety scale (SAS), a self-rated anxiety questionnaire established based on affective symptoms according to diagnostic criteria and not factor analysis studies, was used to measure the level of anxiety in this study. Since that time, numerous nations have adopted the SAS and used it. However, most of the study was conducted in China and Western nations, mostly with members of the general public, healthcare professionals, and medical students (Sundaresan V. et al., 2020).

## Cognitive social theory

This study takes a stand on Bandura’s Social Cognitive Theory (Bandura, 2008), which understands that an individual has the ability to control his behavior. Bandura re-emphasizes that self-efficacy provides an influence in people’s behavior. Believing in one’s abilities positions oneself to adapt to the existing environment. In his study (Bandura, 1986, 1997, 2008; Cioca et al., 2019), he stresses communication and observing others would be a good mechanism to influence behavior patterns. To further elaborate, past experiences and emotions influence behavior patterns. Bandura also mentions that self-efficacy is vital in coping with negative patterns of behavior. Yıldırım and Güler (2020) concluded that negative health behaviors can be prevented through self-efficacy. Likewise, Farooq et al. (2020) noted the same positive influence of self-efficacy. Graf et al. (2021) highlighted clear evidence that there was a lack of studies during COVID-19 on self-efficacy. Furthermore, during the H1N1 pandemic, self-efficacy behaviors were received as positive to curb the consequences of negative behavior pattern showing. Studies by Schwarzer and Renner (2000), Rimal (2001), Luszczynska and Schwarzer (2003), Cho and Lee (2015), Lo et al. (2015), Smith et al. (2016), and Maguire et al. (2019) agreed that self-efficacy has a positive impact on positive behavior exhibition and important for a good behavior outcome. In addition, during the H1N1, it was observed that people groups that exhibited self-efficacy in information-seeking behaviors were able to cope well with the pandemic (Ibuka et al., 2010; Lin et al., 2014). Parallelly, there has been a negative association between self-efficacy and anxiety in studies by Hayes et al. (2004), Butler et al. (2007), Mystakidou et al. (2010), Tan-Kristanto and Kiropoulos (2015), and Mohsin et al. (2022). This was similarly pointed out by Lazarus and DeLongis (1983), Lazarus and Folkman (1984), Compas et al. (1986), Carver et al. (1989), Rudolph and Hammen (1999), Meyer (2001), Connor-Smith and Flachsbart (2007), Amr et al. (2008), Bandura (2008), Lenze and Wetherell (2011), Shamsuddin et al. (2013), Cao et al. (2020), Musa and Aidid (2020), Rahman et al. (2021), Ramli and Mohd (2021), and World Health Organization (2021).

## Self-efficacy

A study by Bandura (1997, 1999, 2008) clearly pointed out that self-efficacy, which is the inner belief to behave in each situation, is crucial to adapt to an environment. Studies by Parsons (2007) and Calinici et al. (2017), involving medical students on online learning, posit that self-efficacy helps to improve knowledge. Self-efficacy is frequently connected to “resilience theory,” which relates to “the process of adapting well in the face of adversity, “trauma, tragedy, threats, or even significant sources of stress” (Newman, 2005). Recent research by Hernández-Padilla et al. (2020) found that self-efficacy is essential to control undesirable behaviors during COVID-19. Kövesdi et al. (2020) also emphasized that self-efficacy plays a protective function during the pandemic. The results of a study conducted in the United Kingdom to gauge the degree of stress and depression among army veterans showed that self-efficacy moderated the connection between posttraumatic stress disorder and battle exposure. Self-efficacy was also widely employed as a moderator (Blackburn and Owens, 2015).

## Methodology

### Conceptual framework

**FIGURE 1:**
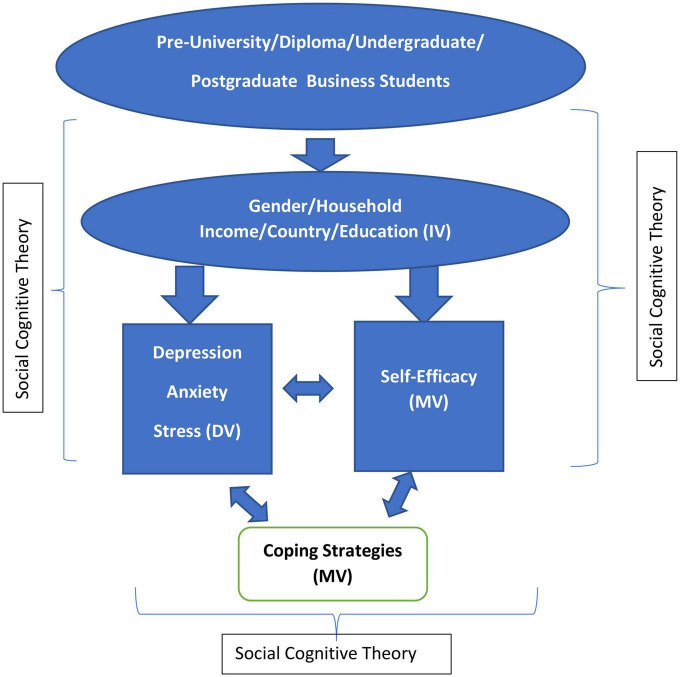
Conceptual framework.

If we focus on cognitive strategy as presented in Figure 1, shows the variables of DAS, self-efficacy, and coping strategies, with the independent variable as gender, level of education, household income, and country, with an underlining social cognitive theory. The framework mirrors the objectives of this study, and it helps to identify the degree of DAS with reference to its independent variables and the influence of self-efficacy and coping strategies.

This study employed a cross-sectional, quantitative research method, with a population size of approximately 10,000 comprising of pre-university/Diploma, Year 1–4 undergraduate business students, and postgraduate business students, where an initial sample size of 506 respondents was gathered from Malaysia, Indonesia, India, China, Southern part of Africa (Southern Africa), and Romania. The respondents were all tertiary students from private institutions of higher education. Data cleansing was undertaken to remove any respondent who has not completed the questionnaire. Therefore, the final useable number of respondents was 397.

Data processed in our research were collected using a Google link form, which was an anonymous survey and a free-willed survey. The link was distributed after obtaining permission from the partner institutions. To qualify as a respondent, the participants must be registered with the institution of higher education in the country of origin before participating in this survey. All information was processed solely for the purpose of this research, and confidentiality was times. In addition, the general self-efficacy scale was used as a moderator with the coping strategies found in the Brief Coping Orientation of Problem Experienced (COPE) by Carver (2013). There were a total 91 questions that the respondents are to answer at their free will.

As per Table 1, many of the respondents were from Malaysia (54.2%), India (40.8%), Southern Africa (2.2%), Indonesia (2%), and China (0.8%). Regarding the gender distribution, 54.2% were women, while males were 45.8% (Table 2).

**TABLE 1:** Respondents by country.

Respondents’ country	Frequencies	Percentage (%)
Malaysia	215	54.2
China	3	0.8
India	162	40.8
Southern Africa	9	2.2
Indonesia	8	2.0
Total	397	100.0

**TABLE 2:** Distribution by gender.

Gender	Frequencies	Percentage (%)
Male	182	45.8
Female	215	54.2
Total	397	100.0

The distribution of the level of education was as follows:

6.3%—Pre-university/Diploma

8.3%—Year 1 Undergraduate

45.1%—Year 2 Undergraduate

32.7%—Year 3 Undergraduate

3.5%—Year 4 Undergraduate

4.1%—Postgraduate.

Therefore, the main proportion of the respondents were from second and third year of Bachler program students from the respective institutions of higher education.

## Instrumentation

The survey questionnaires included country of residence, level of education, gender, and household income. In addition, the Depression, Anxiety, and Stress Scale Short Form (DASS-21), which was developed by Lovibond and Lovibond (1995) and Henry and Crawford (2005), and validated by Vignola and Tucci (2014) served as the basis for the instrument employed in this study (2014).

This is a tripartite model developed using three rudimentary constructions, which are negative affections, specific symptoms of depression, and specific symptoms of areas of anxiety and stress. The scale rates 21 core symptoms ranging from “0” (never) to “3” (almost always). Several studies (Crawford and Henry, 2003; Ng et al., 2007) have confirmed that DASS-21 is a well-established psychometric test. By filling in the survey form, the respondents indicated how much they had experienced (DAS) during the period of pandemic lockdown. This study also included the Coping Scale, which comprises 60 statements with a scale of 1–4, indicating the coping strategies used by the respondents during the same period. Finally, this study employed the general self-efficacy scale (Schwarzer and Jerusalem, 1995) as a mediator to assess the perceive self-efficacy through a four-point scale. The general self-efficacy refers to a respondent’s capability to respond or react during a challenging situation, as noted by Bandura (2008).

The reliability of this study was established using the Cronbach alpha value, where all variables are found to be reliable, that is, all variables registered more than an alpha value of 0.7 (Table 3).

**TABLE 3:** Cronbach’s alpha value.

Variables	Cronbach’s alpha	N of items
Coping strategies	0.938	60
Self-efficacy	0.907	10
Depression	0.890	7
Anxiety	0.849	7
Stress	0.879	7

As for household income distribution, 32.2% were from a household income of less than USD500 per month; 30.2% from household income between USD501 to USD1,000 per month; 14.6% from household income of between USD1,001–USD1,500 per month; 6% from household income of between USD1,501 and USD2,000 per month; and finally 16.9% from household income of above USD2,001 per month.

## Analysis and discussion

SPSS statistical tool was used to analyze the data. The authors began with the descriptive analysis, which is shown in Table 4. The majority of the respondents displayed a “normal” level of DAS; “mild” level of depression as 13%, anxiety as 14%, and stress level as 6%; “moderate” level of depression as 13%; anxiety as 15% and stress level as 3%; “severe” levels were noted on depression and anxiety registering 2 and 3%, respectively. However, only anxiety registered an “extremely severe” as 3%.

**TABLE 4:** Overall descriptive percentages of DAS.

OVERALL	Depression		Anxiety		Stress	
Normal	289	73%	259	65%	364	92%
Mild	50	13%	56	14%	22	5%
Moderate	52	13%	60	15%	11	3%
Severe	6	1%	10	3%		
Extremely severe	0		12	3%		
	397	100%	397	100%	397	100%

From Table 5, it was found that only Malaysian and Indonesian sample sizes were gathered to have registered “severe” and “extremely severe” depression and anxiety levels. There were no such cases in the other jurisdictions. All jurisdictions registered a fairly high level of “normal” levels of DAS.

**TABLE 5:** Descriptive percentage of DAS on country basis.

		Depression	%	Anxiety	%	Stress	%
Malaysia	Severity						
	Normal	137	63.7%	127	59.1%	186	86.5%
	Mild	34	15.8%	30	14.0%	19	8.8%
	Moderate	39	18.1%	38	17.5%	10	4.7%
	Severe	5	2.4%	10	4.7%		
	Extremely severe			10	4.7%		
		215	100.0%	215	100.0%	215	100.0%
Indonesia							
	Severity						
	Normal	4	50.0%	3	37.5%	6	75.0%
	Mild	1	12.5%	1	12.5%	1	12.5%
	Moderate	2	25.0%	2	25.0%	1	12.5%
	Severe	1	12.5%	0	0.0%		
	Extremely severe			2	25.0%		
		8	100.0%	8	100.0%	8	100.0%
India							
	Severity						
	Normal	141	87.0%	119	73.5%	161	99.4%
	Mild	12	7.4%	23	14.2%	1	0.6%
	Moderate	9	5.6%	20	12.3%		0.0%
	Severe				0.0%		
	Extremely severe				0.0%		
		162	100.0%	162	100.0%	162	100.0%
Southern Africa							
	Severity						
	Normal	5	55.6%	8	88.9%	8	88.9%
	Mild	2	22.2%	1	11.1%	1	11.1%
	Moderate	2	22.2%		0.0%		0.0%
	Severe				0.0%		
	Extremely severe				0.0%		
		9	100.0%	9	100.0%	9	100.0%
China							
	Severity						
	Normal	2	66.7%	2	66.7%	3	100.0%
	Mild	1	33.3%	1	33.3%		0.0%
	Moderate				0.0%		0.0%
	Severe				0.0%		
	Extremely severe				0.0%		
		3	100.0%	3	100.0%	3	100.0%

The relationship between depression and coping strategies was analyzed and found that there is a significant relationship between coping and depression, with a *p*-value of less than 0.05.

Using multiple linear regression analysis, it was found that there was a significant relationship between depression and coping and between depression and self-efficacy, both of which registered a *p*-value less than 0.05. Thus, self-efficacy was seen to be a moderating factor in dealing with depression among the respondents. As for anxiety, anxiety and coping strategies were found to be significant, but self-efficacy was found not to be significant with anxiety, hence self-efficacy is not a moderator on the relationship between anxiety and coping strategy. Likewise, for stress, a similar finding was noted, in which self-efficacy was not a moderating factor in stress and coping strategies, contrary to a significant relationship between stress and coping strategy. The results obtained for *Relationship Between Depression and Coping*, *Relationship Between Anxiety and Coping*, *Relationship Between Stress and Coping* are presented in Tables 6–8.

**TABLE 6:** Relationship between depression and coping.

Depression		Decision
Pearson correlation	0.194	Supported
Sig. (2-tailed)	0.000	
N	397	

Subsequently, the relationship between anxiety and coping strategies was found to be significant as *p*-value was below 0.05.

**TABLE 7:** Relationship between anxiety and coping.

Anxiety		Decision
Pearson correlation	0.265	Supported
Sig. (2-tailed)	0.000	
N	397	

The relationship between stress and coping was also found to be significant, where *p*-value was below 0.05.

**TABLE 8:** Relationship between stress and coping.

Stress		Decision
Pearson correlation	0.246	Supported
Sig. (2-tailed)	0.000	
N	397	

Using the independent *t*-test, it was found that gender had no difference in the mean coping strategies in mitigating the level of DAS for both males and females. As for level of education, by using ANOVA, there was no significant relationship between level of education and coping strategy. However, on deeper scrutinization, the following tables depict that all levels of education recorded that there were experiencing depression in one way or other. There was a significant increase in number of respondents in the categories of *“Applied to me to a considerable degree”* and *“Applied to me very much”* as per Tables 9–13 (highlighted in bold)

**TABLE 9:** Current level of education * cmDepression crosstabulation.

Count					
		cmDepression			Total
		Did not apply to me at all	Applied to me to a considerable degree	Applied to me very much	
Current level of education	Pre-University/Foundation/Diploma/IB	5	16	4	25
	Year 1—Undergraduate	13	16	4	33
	Year 2—Undergraduate	43	**111**	**25**	179
	Year 3—Undergraduate	32	**68**	**30**	130
	Year 4—Undergraduate	1	6	7	14
	Postgraduate	5	8	3	16
Total		99	225	73	397

Both the Year 2 and Year 3 undergraduate levels registered (111 + 25 + 68 + 30) approximately 59% of the total sample size that depression did affect them during the pandemic. Likewise, the level of anxiety was recorded similarly for the rest of the levels.

**TABLE 10:** Current level of education * cmAnxiety crosstabulation.

Count					
		cmAnxiety			Total
		Did not apply to me at all	Applied to me to a considerable degree	Applied to me very much	
Current level of education	Pre-University/Foundation/Diploma/IB	5	12	8	25
	Year 1—Undergraduate	16	16	1	33
	Year 2—Undergraduate	47	120	12	179
	Year 3—Undergraduate	42	64	24	130
	Year 4—Undergraduate	2	8	4	14
	Postgraduate	4	9	3	16
Total		116	229	52	397

**TABLE 11:** Current level of education * cmStress crosstabulation.

Count					
		cmStress			Total
		Did not apply to me at all	Applied to me to a considerable degree	Applied to me very much	
Current level of education	Pre-University/Foundation/Diploma/IB	4	12	9	25
	Year 1—Undergraduate	15	17	1	33
	Year 2—Undergraduate	40	120	19	179
	Year 3—Undergraduate	27	73	30	130
	Year 4—Undergraduate	1	8	5	14
	Postgraduate	3	8	5	16
Total		90	238	69	397

**TABLE 12:** Current level of education * cmSelf crosstabulation.

Count					
		cmSelf			Total
		Did not apply to me at all	Applied to me to a considerable degree	Applied to me very much	
Current level of education	Pre-University/Foundation/Diploma/IB	1	17	7	25
	Year 1—Undergraduate	2	18	13	33
	Year 2—Undergraduate	3	67	109	179
	Year 3—Undergraduate	4	47	79	130
	Year 4—Undergraduate	0	6	8	14
	Postgraduate	0	6	10	16
Total		10	161	226	397

In the area of self-efficacy, Year 2 and Year 3 undergraduates mirrored better self-efficacy than the other levels of education. These two categories (67 + 109 + 47 + 79) represent 76% of the entire sample size. Likewise, a similar trend was noted with the pre-university, Year 1, Year 4, and postgraduate levels. It means that the self-efficacy evaluation was relevant.

**TABLE 13:** Current level of education * cmCope crosstabulation.

Count					
		cmCope			Total
		Did not apply to me at all	Applied to me to a considerable degree	Applied to me very much	
Current level of education	Pre-University/Foundation/Diploma/IB	0	24	1	25
	Year 1—Undergraduate	2	26	5	33
	Year 2—Undergraduate	1	133	45	179
	Year 3—Undergraduate	3	89	38	130
	Year 4—Undergraduate	0	11	3	14
	Postgraduate	0	11	5	16
Total		6	294	97	397

As for coping strategies, Year 2 and Year 3 undergraduates registered higher managing capabilities with DAS. These two categories (133 + 45 + 89 + 38) represent 76.8% of the entire sample size. The other levels were finding coping to be useful as well and relevant.

The pandemic has affected the level of DAS among tertiary students (Lyon and Matson, 2020). Our data show the level of stress in Malaysia and in selected emerging economies. While much of the sample originates from Malaysia and India, this study provides a substantial indication on the level of DAS and the coping strategies with the general self-efficacy as a moderator. The findings showed that due to the coping strategies employed, and the self-efficacy applied, the students were able to manage DAS quite effectively, unlike many studies such as Pan et al. (2020) and Zhai and Du (2020), where levels of DAS were rather significant. In another study by Al Omari et al. (2020) involving 1,057 respondents noted the level of DAS as 57, 40.5, and 38.2%, respectively. Likewise, studies by Fawaz and Samaha, 2021) found that among a sample size of 520 Lebanese tertiary students, 15.5% had moderate depressive symptoms while 30.5% showed anxiety symptoms.

The findings of this study are that while DAS is well managed across the sample size, there were significant findings which are as follows.

a) **The household income of USD 1,000 and below registered a much significant DAS than the other groups of household income**. This is in tandem with the finding from Ettman et al. (2020), in which the Patient Health Questionnaire was used, and it was reported among the 1,470 US adults, individuals with a lower level of income registered a higher level of depression.
b) **All levels of education registered that both self-efficacy and coping strategies were applicable to them.** While the sample size is skewed toward Year 2 and Year 3, it also denotes that the pressure of studies and challenges are a reality during these levels of education. It also raises the need for more counseling sessions for all of this group of students. A greater reach is required to maintain the level of coping and self-efficacy. This is also in line with studies by Jiang et al. (2021) which highlighted that many Malaysian higher learning institutions have an encouraging mechanism to deal with students with DAS, coupled with clear information channels to disseminate COVID-19-related news and updates.
c) **Generally, there was no significance of DAS regarding country, gender, level of education, and household income**. The sample represents students from private tertiary IHLs. This is contrary to studies by Ibrahim et al. (2013) and Rotenstein et al. (2016). Also, it is contrary to the studies by Baloran (2020), Capone et al. (2020), Lai et al. (2020), Nurunnabi et al. (2020), Wathelet et al. (2020), Almomani et al. (2021), Jiang et al. (2021), Mekonen et al. (2021), Patias et al. (2021), Sood and Sharma (2021), and Villani et al. (2021).
d) **Self-efficacy has no significance in its relationship with stress and anxiety, unlike depression**. In other words, both stress and anxiety are well manageable with coping strategies. It showed that most of these millennials were able to cope well during the COVID-19 pandemic. On analyzing the 10 questions on the general self-efficacy scale, it was noted that 59.6% out of the 3,970 responses (10 questions × 397 respondents) registered a scale of 3 (moderately true) and 4 (exactly true). Among the 10 questions, the questions on “*I can always manage to solve difficult problems if I try hard enough”* scored 70.5% and *“I can solve most problems if I invest the necessary effort”* scored 72.3%, respectively. This proves that self-efficacy has been well applied in this sample (Table 14).

**TABLE 14:** General self-efficacy.

	Moderately true (3)	Exactly true (4)	Total	Overall %
1. If I work hard enough, I can always find a method to fix an issue.	171	109	280	70.5%
2. Even if someone opposes me, I can find ways and means to achieve my goals.	151	64	215	54.2%
3. I can stick to my plans and achieve my objectives with ease.	116	102	218	54.9%
4. I have faith in my ability to handle unforeseen circumstances well.	156	74	230	57.9%
5. Because I’m resourceful, I can deal with unforeseen circumstances.	125	55	180	45.3%
6. If I put in the necessary effort, I can solve many difficulties.	161	126	287	72.3%
7. I can maintain my composure in the face of challenges because I have coping mechanisms.	143	69	212	53.4%
8. I can generally come up with a few solutions when I’m faced with an issue.	156	72	228	57.4%
9. I can typically come up with a solution when I’m in trouble.	147	125	272	68.5%
10. Usually, I am able to handle any situation.	159	85	244	61.5%

From Table 14, the authors included scale 3 “moderately true” and 4 “exactly true” to intentionally gauge the degree of positivity in self-efficacy. Summary of the analysis is presented in Table 15. It was found all the nine areas shown recorded a percentage of more than 50% except for Scale No. 5, which recorded a 45.3%. This reflects that the sample has the inner belief that they can manage despite the COVID-19 pandemic. The findings are parallel to studies by Azila-Gbettor et al. (2021), which state that self-efficacy is much stronger during periods of uncertainty and ambiguity. Similarly, Yıldırım and Güler (2020) in the Turkish adult sample noted that self-efficacy has a positive impact during the COVID-19 outbreak. Similarly, Tabernero et al. (2020) noted that self-efficacy correlated with the behavior exhibited by COVID-19 Spanish people. With the help of the generalized self-efficacy scale (GSES), Wang et al. (2020) discovered a positive correlation between self-efficacy and professional identity. This reflects on the results obtained in DAS (Table 5) above, which recorded a high level of the “normal” scale. This reveals that this millennial sample has the belief and confidence that they could handle the challenges thrown at them by the pandemic.

**TABLE 15:** Summary of the analysis.

	Questions	Average percentile
Problem-focused coping		51.9%
Active response	5, 31, 42, 56	57%
Planning	18, 99, 39, 56	57.6%
The suppression of competitors activities	16, 31, 42, 55	48.7%
Utilizing restraint	10, 22, 41, 49	46.6%
Seeking social support for the use of instruments	4, 14, 30, 45	49.6%
Emotion-focused coping		48.7%
Requesting social support since you’re feeling down	11, 23, 34, 52	43.0%
Positive revision and development	1, 29, 38, 59	66.6%
Acceptance	13, 21, 44, 54	60.1%
Denial	6, 27, 40, 57	29.1%
Utilizing religion	7, 18, 48, 60	44.9%
Less useful		42.4%
Attention to and expression of emotions	3, 17, 28, 46	45.2%
Conduct disengagement	9, 24, 37, 51	27.5%
Disengagement from reality	2, 16, 31, 43	54.5%
Two additional scales		23.2%
Humor	8, 20, 36, 50	35%
Substance use	12, 26, 35, 53	11.3%

1. While the majority of DAS was on a “normal” scale, but this study **revealed that 26% were found in the “mild” and “moderate” scale for depression, while 29% under anxiety scale and 9% on stress scale. Parallelly, it was noted that “severe” was 2% for depression and 3% for anxiety, while “extremely severe” was at 3% for Anxiety**. This means even with coping strategies, close to one-third are exhibiting a concerned level of depression and anxiety. This is noteworthy, and IHL must act to mitigate the progress of these cases.

Tabulating the 22 received statements, registering more than 50% with that in Table 5, shows that in the coping strategies, the sample was able to apply problem-focused coping strategies, mainly, “active coping” and “planning” strategies, with a tinge of “suppression of competing activities,” “restraint coping,” and “seeking social support for instruments reasons.” Under the COPE model on emotional-focused coping, this study found that the sample applied “positive reinterpretation and growth strategy coupled with acceptance strategy.” It was also found a relatively small percentile in “less useful” coping strategies especially in “mental disengagement.” It was also found that “humor” and “substance use” were present but minimal. **This reveals that the sample can cope well; hence, the resulting DAS score for “normal” scale was relatively higher.**

## Conclusion and recommendations

The higher education institutions and universities presented in this study provided satisfactory support regarding the challenges of data collection process, determined by unstable internet connectivity. However, this exploratory study provided a good indication of the causality of the actions taken to strengthen and maintain momentum to help students manage DAS. This study shows that the pandemic period had effects on Students’ health. This research provided insight that, contrary to other research conducted in the field, students from our sample, particularly from Malaysia and India, have shown success in managing DAS. The mental health of the students included in our research has provided a glimpse of how millennials are coping well, although much of the traditional face-to-face interactions and teaching have been restructured online or obscured. In this new norm of remote learning or e-learning, constant humanistic values are required to monitor students with a well-balanced ecosystem within the IHL. While there were a small portion of “severe” and “extremely severe” cases noted in both Malaysia and Indonesia, it cannot be ignored that constant effort to address DAS and effective counseling is crucial. Jiang et al. (2021) stressed that in this ecosystem, it is not only the responsibility of the management of these higher learning institutions, but all stakeholders, including lecturers, should be alert when interacting with students from the millennial generation. This study agrees with the view of Lyon and Matson (2020) in that coping strategies should be disseminated and educated to students for their wellbeing and such humanistic endeavors are the key for wellbeing during the COVID-19 pandemic.

Considering the limitations of our research, we can affirm that the sample size is rather small compared to the population, and equal samples from each category were not representative. Therefore, future research should consider a larger sample from other nations to have a greater revelation of the findings. Also, peer support data should be included, as millennials would have a better interaction with their peers, as noted by Brown and Larson (2009). A deeper analysis of Brief COPE and self-efficacy with a larger sample would reflect a greater understanding to support future research. Another limitation of this study refers to the distribution of respondents. The students involved in the current study live in Malaysia, China, India, Southern Africa, and Indonesia. These territories are representative by the large size of the students. This study can be extended to the level of Europe or another region.

Finally, higher learning rural institutions must be integrated to better understand their DAS with coping strategies and the self-efficacy approach.

## Data availability statement

The original contributions presented in this study are included in the article/supplementary material, further inquiries can be directed to the corresponding author.

## Ethics statement

The studies involving human participants were reviewed and approved by Sunway University’s Research Ethics Approval—Ref: SUREC 2021/047. The patients/participants provided their written informed consent to participate in this study.

## Author contributions

All authors listed have made a substantial, direct, and intellectual contribution to the work, and approved it for publication.
